# The pros and cons of the PCC staging system to guide surgical resectability and prognosis

**DOI:** 10.7150/jca.76696

**Published:** 2022-10-09

**Authors:** Pei Liu, Yinghui Song, Kashif shakoor, Chuang Peng, Sulai Liu

**Affiliations:** 1 Department of Hepatobiliary Surgery, Hunan Provincial People's Hospital, The First Affiliated Hospital of Hunan Normal University; Changsha 410005, China.; 2 Central Laboratory of Hunan Provincial People's Hospital, The First Affiliated Hospital of Hunan Normal University, Changsha, Hunan 410005, P.R. China.

**Keywords:** Perihilar cholangiocarcinoma, types, staging

## Abstract

Perihilar cholangiocarcinoma (PCC) is a malignant mass originating from the bile ducts. There is currently no unified treatment plan, and there are various treatment methods applied in clinical practice, as well as several different staging and typing systems to guide resectability, prognosis and survival prediction. The choice of treatment for PCC is closely related to the stage of the tumor. Accurate preoperative staging is necessary for correct resectability assessment and the selection of a reasonable treatment plan and surgical method; similarly, accurate postoperative pathological staging is necessary to guide further treatment and judgment of the patient's prognosis. A universally accepted staging system facilitates the comparison of cases between different centers, but there is much debate about the classification and staging of PCC. At present, the existing staging systems include the Bismuth-Corlette classification, AJCC/UICC TNM staging, modified T staging, Gazzaniga staging, JSBS staging, and Mayo staging. Each system has advantages, but there is no comprehensive guide for tumor resectability, prognosis, and survival. In this paper, the pros and cons of the different systems for staging PCC in terms of resectability, prognosis and survival prediction are discussed.

## Introduction

Cholangiocarcinoma (CCA) is an infrequent malignant tumor that develops from the bile duct epithelium (bile duct, BD) and can arise in any part of the biliary tract 1. It has been reported that the overall incidence of CCA has been increasing recently 2-5. CCA can be divided into intrahepatic cholangiocarcinoma (ICC) and extrahepatic cholangiocarcinoma (ECC) according to its anatomical location, and ECC can be further subdivided into hilar and distal. CCA is highly lethal, with a 5-year survival rate of only 10% since the 1980s 6. Presently identified risk factors for cholangiocarcinoma include primary sclerosis cholangitis, liver fluke infection, inherited fibrous polycystic liver, cholangial adenoma and papillomatosis, hepatolithiasis, chemical carcinogens such as nitrosamines, chronic viral hepatitis, cirrhosis, chronic nonalcoholic liver disease and obesity 7.

Extrahepatic cholangiocarcinoma (ECC) can be classified as perihilar (involving the junction of the bile ducts at the hilum) or distal (the middle and lower half of the bile duct, usually located in the head of the pancreas). These two subtypes differ in genetics, clinical presentation, management, and patient prognosis. Therefore, each subtype requires its own staging system to predict surgical resectability and survival and to guide treatment. Approximately 60 percent of CCAs are located in the perihilar region, 30 percent occur in the middle and lower bile ducts, and 6 to 10 percent are intrahepatic cholangiocarcinoma 8, 9. Perihilar cholangiocarcinoma (PCC, also known as Klatskin tumor) is the most common subtype. The concept of PCC began to attract attention after it was first reported by Klatskin in 1965 10.

Surgical resection is the mainstay of curative treatment of PCC, but less than 25% of patients can be treated with radical resection at the time of diagnosis 11. In a study of 257 patients with PCC who underwent radical resection, the five-year survival rate was 19% 12. Liver transplantation plus neoadjuvant therapy has been proposed as an alternative to resection in PCC patients 13. Patients with hilar cholangiocarcinoma treated with neoadjuvant therapy and liver transplantation at 12 medical centers in the USA had a survival rate of 65%, which may seem impressive. However, this was accomplished with extremely strict case selection 14.

There are limited treatment options for patients who are not eligible for resection or transplantation. The current first-line regimen of chemotherapy is gemcitabine combined with cisplatin, which delays disease progression from 5 months to 8 months compared with the previously applied single-agent chemotherapy. However, the overall survival only increases from 8.1 months to 11.7 months 15. More recent studies have shown that with the development of clinical oncology and the completion of various clinical drug trials, immunotherapy may be a feasible way to treat cholangiocarcinoma in the future 16.

Therefore, new treatments to improve the outcomes of patients with PCC are urgently needed. The subtype and stage of PCC are of great clinical significance because they can guide the evaluation of tumor resectability and predict postoperative outcomes. At present, the main staging methods are as follows: Bismuth-Corlette classification, TNM staging of AJCC/UICC, modified T staging, Gazzaniga staging, JSBS staging, and Mayo staging (see Table 1 for details). This article will review the existing staging methods, focusing on their pros and cons for judging the feasibility of PCC surgical resection and predicting survival.

## Bismuth-Corlette Type

In 1975, Bismuth et al. proposed a system of classification of PCC 17 and revised it in 1992 18. The Bismuth-Corlette classification is based on the location of the tumor in the bile duct and divides it into 4 types (Table 2). This system is the most widely used PCC classification system because it is not complicated, is clear and easy to differentiate and is consistent with the tumor's pathological and behavioral characteristics. The different types correspond to different surgical approaches to resection.

Bismuth retrospectively studied 122 patients who underwent surgery from 1960 to 1990; 23 patients underwent surgical resection, of whom 10 (43%) underwent local resection (cholecystectomy). Hepatectomy was performed in 13 patients (57%) due to tumor invasion into the secondary bile duct: extended right hepatectomy (3 cases), right hepatectomy (1 case), extended left hepatectomy (6 cases), left hepatectomy surgery (2 cases) and left lobectomy (1 case).

In this study, Bismuth noted that for type I tumors, only resection of the locally diseased bile duct was needed, i.e., extrahepatic bile duct resection, cholangiojejunostomy, and duodenal ligament lymph node dissection. Combined caudate lobe resection is often required for type II lesions because of the inevitable involvement of the bile duct draining the caudate lobe (segment I). Moreover, segment IV excision is sometimes required for larger tumors involving segment IV.

Local resection of type III disease has difficulty achieving safe margins, so it is necessary to resect the liver segment drained by the bile duct, that is, type IIIa resection of the right half of the liver or enlarged right liver and combined resection of the caudate lobe, type IIIb resection of the left half of the liver or enlarged left liver hemihepatic combined with caudate lobectomy.

For type IV lesions, liver transplantation or central liver resection/right trisectionectomy/left trisectionectomy is recommended, with routine lymph node dissection and reconstruction of tumor-invading blood vessels. For patients with preoperative cholangitis or poor liver function, percutaneous transhepatic bile duct drainage is suggested to improve liver function and prevent postoperative deterioration of liver function 18. For patients with Bismuth type II, III, or IV tumors, combined caudate lobectomy can remarkably improve the R0 resection margin rate 19, 20.

Recently, neoadjuvant chemotherapy combined with liver transplantation has made great progress in unresectable Bismuth type III PCC patients, which can significantly improve their 5-year survival rate 13. For the selection of Bismuth type III and IV surgical approaches, studies have shown that the hilar approach may be superior to the conventional approach because the hilar approach can obtain a higher negative margin rate, higher survival rate and lower surgical mortality rate 21-23.

Whether open surgery or endoscopic surgery is better is still inconclusive. Our previous study showed that the application of laparoscopic radical resection for III/IV hilar cholangiocarcinoma is safe and feasible and has good short-term efficacy with adequate preoperative evaluation, appropriate case selection, and a precise operative strategy 24. Other research shows that there is no difference in the interim efficacy between the two surgical methods, but open surgery has superior long-term efficacy 25.

Corroborative evidence has shown that the tumor location does not have a significant effect on patient survival. However, these tumors exhibit varying degrees of propensity to invade adjacent structures depending on their site of origin, which has implications for the role of surgery and the long-term prognosis 26. The latest studies have shown that intrahepatic cholangiocarcinoma involving the hepatic hilum has a more aggressive biological behavior and a worse prognosis than PCC 27-29. Because it is very difficult to identify the primary location of a hilar mass on the basis of clinical and imaging examinations before surgery, we often confuse the two with each other. There are also reports in the literature that there are cases of jaundice caused by the growth of hepatocellular carcinoma compressing the hepatic hilum 30.

Based on all of the above, we believe that the shortcomings of the Bismuth-Corlette classification are so obvious that it can be considered an imperfect stratification even when evaluating masses located around the hepatic hilum. At the same time, because this stratification system simply evaluates the tumor growth position in the bile duct and does not consider vascular invasion, lymph node metastasis, tumor size, etc., it has serious limitations in prognostication 31, 32.

## TNM staging

The TNM staging system (Table 3) was first publicized by the American Joint Cancer Society (AJCC) in 1977. This classification system considers the size of the primary tumor (T), the number of regional lymph node metastases (N), and the size and extent of distant metastases (M). It is currently the most widely used clinical staging system and has become the basis of tumor treatment. The latest version is the 8^th^ edition 33.

The AJCC staging system of PCC has undergone three extraordinary updates in recent years: in its 6th edition in 2003, all patients with extrahepatic bile duct tumors were included in the grading 34. The 7^th^ edition in 2010 was the first system to differentiate between perihilar (proximal) and distal cholangiocarcinoma 35. In the 8th edition, Japanese scholars Ebata et al. 29, 36 showed that the survival rate of Bismuth IV was comparable to that of Bismuth I-III for patients with pN0M0 tumors undergoing R0 resection. Therefore, the T4 disease category in the 8th edition does not include bilateral secondary bile duct invasion (Bismuth type IV). In addition, T4 tumors were reduced from IVA to IIIB because the resection of these tumors is feasible in large specialized centers capable of extended liver resection, combined vascular resection, and reconstruction, which also marks technical progress in the surgical treatment of PCC. In the 8^th^ edition, the lymph node staging (class N) was changed for patients with PCC, gallbladder, distal bile duct, ampulla, and pancreas, with N1 defined as 1 to 3 metastatic lymph nodes and N2 as 4 or more metastatic lymph nodes. Metastatic lymph node classification results based on these numbers allow for better prognostic stratification 37, 38.

An optimal staging system is supposed to provide information about prognosis, guide treatment, and allow for comparisons with different staging systems. However, the current TNM staging system for PCC does not meet these specifications; studies have found that the 8^th^ edition of the AJCC staging system does not provide a better prognostic ability for PCC patients than the 7^th^ edition, and its ability to predict patient survival remains poor and needs to be improved 39-41. In addition, a meta-analysis found that some factors that specifically affected the overall survival of PCC patients, including microvascular invasion, peripheral nerve invasion and other factors, were not considered in the AJCC staging 42.

Therefore, TNM staging is still restricted in guiding judgments about the feasibility of resection and prognostication, and it needs to be improved.

## Modified T staging

The widely used Bismuth‒Corlette classification and TNM staging cannot be precisely used for the preoperative evaluation of tumor resectability. The MSKCC T staging (Table 4) was first proposed by Burke 43 in 1998 and further revised in 2001 44. This T staging system is mainly used for the preoperative evaluation of patients by pointing out that the long-term survival of PCC patients is heavily dependent on radical resection. The possibility of achieving radical resection requires examining all factors related to local tumor extent. Therefore, it includes 3 factors: the extent of tumor invasion of the bile duct, whether it has invaded the portal vein, and whether the liver lobe is atrophied. This classification does not consider factors such as arterial involvement, lymph node and distant metastasis, and it does not include an authoritative definition of liver atrophy, which seriously limits the clinical application of this classification.

Zaydfudim et al. 45 sequentially analyzed the survival of 80 patients with Bismuth type III PCC who underwent surgical treatment using the 7th edition of AJCC and MSKCC T staging and found that there was no association between either of the staging methods and recurrence-free survival; only MSKCC T staging corresponded with overall survival. However, some authors believe that MSKCC staging does not have any prognostic value 46. Stefan Buettner et al. 47 analyzed the data of 407 patients who underwent surgery and concluded that MSKCC staging and its nomogram have poor predictive capability for the long-term survival of PCC patients. Hemming et al. 48 and Ito et al. 49 showed that the modified T staging system was not associated with PCC prognosis or survival time.

Therefore, this staging system can be used to analyze the resectability of the mass before surgery, but it has limited guiding significance for the prognosis and survival evaluation of postoperative patients.

## Gazzaniga Staging

Gazzaniga 50 first proposed Gazzaniga staging (Table 5) in 1985. It is a staging system based on the degree of bile duct and vascular invasion. The classification only considers the invasion of the tumor to the liver blood vessels, and the site of origin of the PCC, lymph node status, and distant metastasis are not included in the staging criteria. Gazzaniga et al. studied 159 HCCA patients classified by these criteria. A total of 75 patients underwent surgical treatment (resectable rate 47.2%), and 46 patients underwent radical resection (radical resection rate 28.9%). The study showed that the 5-year survival rate of patients undergoing radical resection was 17.5%, and the median survival time was 19 months 51. Studies have shown that for predicting the overall survival of PCC patients, a nomogram established by the Shanghai Oriental Hepatobiliary Hospital and validated internally and externally is superior to Gazzaniga staging 52.

This staging system is of little significance for the selection of preoperative surgical methods and the evaluation of the prognosis, so this classification system is rarely used in clinical practice.

## JSHBPS Staging

The first edition of the Japanese Classification of Biliary Carcinoma (JC) organized by the Japanese Society for Biliary Surgery (JSBS) (titled “General Guidelines for the Study of Surgical Pathology in Biliary Carcinoma”) was published in 1981 53. Since 1981, JSBS has revised the staging system 4 times. The English version was published on the basis of the 4th and 5^th^ Japanese editions in 2001 and 2004, respectively, but it is only applicable to patients who underwent surgery. To facilitate integration with the cancer staging systems of various international research centers, the 6th edition of the staging system (Table 6) also began to be based on the size of the primary tumor (T), the number of regional lymph node metastases (N), and the presence of distant metastases (M), similar to the TNM staging system 54.

A study comparing JC staging with UICC/AJCC pointed out that the differences between the two for biliary tract tumors are mainly reflected in PCC and ampullary cancer 55. The definition of PCC in the JSHBPS varies, and in the UICC/AJCC staging system, PCC is anatomically defined as a tumor in the extrahepatic bile duct tree proximal to the origin of the cystic duct. The new 6^th^ edition staging has an ambiguous definition of the extrahepatic bile duct tree, and it is difficult or even impossible to distinguish intrahepatic cholangiocarcinoma from PCC in advanced tumors. Nakeeb et al. 56 simply defined hilar tumors as tumors involving or requiring resection of hepatic duct bifurcations, even those with a major intrahepatic component. This definition is too broad to include overt intrahepatic cholangiocarcinoma with invasive lesions in the perihilar bile ducts.

Therefore, in the 2^nd^ edition 57, the proximal border of the perihilar bile duct is marked by the junction of the right anterior and posterior bile ducts and the junction of the left and right bile ducts. Hilar cholangiocarcinoma is defined as a tumor that occurs in the hilar bile duct. Because of the frequent anatomical variation of the biliary system, this definition is conflicting; therefore, in the 6th edition, the reference landmark for the proximal border of the perihilar bile duct was changed to the portal venous system. The umbilical part of the left portal vein branch is regarded as the “U” point, and the origin of the right posterior branch of the portal vein is regarded as the “P” point, as the proximal limit of the hilar bile duct 57.

In the 6^th^ edition, tumors invading the roots of bilateral secondary bile ducts (Bismuth type IV tumors) were subdivided into T4a on T staging. The reason for this is that data from a representative large hospital in Japan showed that the survival rates of patients with Bismuth‒Corlette I-III and IV tumors were similar when pN0M0 patients undergoing R0 resection were studied 28. Tumors that invaded the main portal vein, inferior mesenteric vein or inferior vena cava, and gallbladder cancer that invaded extrahepatic bile ducts were classified as T3b; in N staging, the scope of the regional lymph nodes was limited to Group 8, 12 and 13a lymph nodes, and lymph node metastasis beyond this region was regarded as distant metastasis; in M staging, cases with positive peritoneal lavage cytology were regarded as M0 with the code name “Pcy1” added to distinguish it from other M0 cases.

In the new JC, the original traditional classification of margins (Cur A/B/C) was removed, and the residual tumor after surgical resection was classified according to R, which is consistent with the UICC/AJCC staging system. On the issue of surgical margins and tumor remnants, the 6th edition of JC staging is consistent with UICC/AJCC staging, but the "R1" margin type in UICC/AJCC staging does not take into account the microscopic involvement of ductal resection margins. It has been reported^58^, ^59^ that patients with residual intraepithelial tumors at the margin of ductal resection have better survival rates than patients with invasive carcinoma and similar survival rates to patients without residual tumors. Therefore, based on these findings, residual intraepithelial tumors should be classified as R1 but should be supplemented with a 'cis' marker to differentiate them from residual invasive tumors.

In a study comparing the practicality of the TNM and JC staging systems, 128 patients who underwent surgical resection were retrospectively classified, and the survival curves of TNM stages IIB and IV were more similar than those of IIA or III, and the IIB and stage IV survival rates were significantly lower than the other stages. There were significant differences between JSBS stages I and III, IV A and IVB, II and IV A/IVB, and III and IV A/IVB, indicating that JSBS staging is better for patient prognosis than TNM staging 60.

Because this classification is mainly used to evaluate the survival of patients who can be surgically resected, the prognosis of patients with unresectable tumors cannot be evaluated yet, and it is necessary to continue to collect clinical data and improve it.

## Mayo Staging

Most patients with PCC have no possibility of surgical treatment at the time of diagnosis 11, but most of the cholangiocarcinoma classification and staging systems are designed to evaluate the feasibility of surgical resectability and the surgical approach or to evaluate the prognosis after surgery. The goal of Mayo staging is to provide a clinical staging system for PCC that will predict the outcomes of all PCC patients and help stratify PCC patients for inclusion in clinical trials 29. In 2014, Chaiteerakij 61 and others retrospectively analyzed the clinical data of 413 PCC patients who were treated at the Mayo Clinic in the United States from 2002 to 2010 and used this information to develop Mayo staging (Table 7). Mayo staging considers the primary tumor, tumor size and number, vascular invasion, lymph node and peritoneal metastasis, and carbohydrate antigen 19-9 (CA19-9) level to stratify patients into a 4-stage staging system. The median survival times of patients with stage Ⅰ, Ⅱ, Ⅲ and Ⅳ disease were 48.6 months, 21.8 months, 8.6 months and 2.8 months, respectively (P<0.0001). Compared with the TNM staging system, this staging system has superior value for predicting survival. The validation study conducted by Coelen et al. 62 also yielded equivalent results, showing that the Mayo staging system is not useful for judging tumor resectability but has superiority in predicting survival. The results of a study on the predictive accuracy of Mayo staging in an Asian population showed that Mayo staging had good performance in predicting the survival of patients with early and advanced stages but limited performance in distinguishing the prognosis of patients with intermediate stages 63.

Therefore, Mayo staging has advantages in predicting patient survival, but there are no large sample data to show the value of Mayo staging in the preoperative assessment of tumor resectability. At the same time, because this staging is only based on single-center study data, it still needs to be validated in additional centers. More research data will be required for further verification.

## Summary

At present, surgical treatment provides the only possibility for curing PCC. PCC arises in the hilum hepatis and the anatomical structure of the hilum hepatis is complex. There are often variations in blood vessels and bile ducts, resulting in difficult resection of the tumor, high surgical risk, low radical resection rate and a poor prognosis. Surgery often requires the removal of a large amount of liver, dissection of the hepatoduodenal ligament and lymph nodes, and reconstruction of arteries/veins and the bilioenteric internal drainage. Therefore, accurate preoperative evaluation of the feasibility of resection and surgical method selection are of paramount importance. Nevertheless, most patients have already lost the opportunity for surgery at the time of diagnosis. Therefore, the choice of treatment mode for PCC is closely related to tumor staging, and a staging system with high clinical application value should have clear objectives, be easy to apply, and be readily comparable to other staging systems. It should provide comprehensive guidance for tumor resection evaluation, surgical procedure selection, comprehensive postoperative treatment and survival prediction. However, due to the complexity and difficulty of PCC treatment, there is no unified classification or staging to provide at present. Each country and even each medical center has proposed their own classification and staging system to guide the diagnosis and treatment of PCC, which also indirectly illustrates the complexity and difficulty of the treatment of PCC.

Therefore, as hepatobiliary surgeons, it is helpful for us to be familiar with the various classification and staging systems used for the clinical diagnosis and treatment of PCC. In the staging systems mentioned above, the Bismuth-Corlette classification can be used to guide the selection of surgical options; MSKCC T staging is suitable for evaluating the resectability of tumors; the Mayo staging system is mainly useful for predicting patient survival; and the TNM staging of AJCC/UICC and JSBS can be used to guide postoperative treatment and judge the prognosis. Gazzaniga staging does not take into account the location of the PCC, lymph node status, distant metastasis or other factors, and thus it needs to be improved.

Therefore, the 7^th^ edition of JSBS staging should be used for the evaluation of resectability. If the patient has resectable disease, the surgical method can be selected according to the Bismuth-Corlette classification. Since pathological specimens can be obtained after surgery, TNM staging is the best choice for prognostication. Mayo staging can be used for prognostication of patients who cannot be treated surgically.

## Figures and Tables

**Table 1:** Classification of PCC staging system

Type	Advantages	Disadvantages
Bismuth-Corlette	Guidance for surgical methods	Poor Evaluation of resectability and prognosis for postoperative
TNM Staging	Widely used as the basis of cancer treatment	Poor prediction for postoperative survival time
MSKCC T Staging	Good evaluation of tumor resectability	The definition of hepatic atrophy is unclear
Gazzaniga Staging	Easy to understand	Poor Evaluation of resectability and prognosis for postoperative
JSHBPS Staging	Using a novel hilar demarcation marker to guide tumor resectability	Limited prognostic significance for unresectable masses
Mayo Staging	Good predictor of survival in unresectable masses.	Only for the evaluation of survival in patients with unresectable tumors

**Table 2:** Bismuth-corlette typing and surgical scheme

Staging	Definition	Surgical resection site
I	Tumor in common hepatic duct	Extrahepatic bile duct, duodenal ligament lymph node
II	Tumor with confluence involvement	Type I + caudate lobe, sometimes IV (lager tumor).
IIIa	Tumor with the confluence and right hepatic duct involvement	Right hemi-liver/enlargement of the right hemi-liver+caudate lobe
IIIb	Tumor with the confluence and left hepatic duct involvement	Left liver/enlargement of the left liver + caudate lobe
IV	Tumor invasion of bilateral intrahepatic secondary bile ducts	Liver transplantati-on/middle lobe/right three-zone lobe/left three-zone lobe, lymph node+reconstruction of the invaded vessels.

**Table 3:** TNM staging

Staging	0	I	II	IIIA	IIIB	IIIC	IVA	IVB
T	TIS	1	2a-b	3	4	random	random	random
N	0	0	0	0	0	1	2	random
M	0	0	0	0	0	0	0	1

Note: Tumor size (T); TX: The primary tumor cannot be evaluated; T0: No evidence of primary tumor; Tis: Carcinoma *in situ*/Severe dysplasia; T1: Limited to bile ducts, reaching muscularis or fibrous tissue; T2a: Beyond the bile duct wall to the surrounding adipose tissue; T2b: Invasion of adjacent liver parenchyma; T3: Invasion of one branch of the portal vein or hepatic artery; T4: Invasion of the portal vein or its bilateral branches, or common hepatic artery, or bilateral secondary bile ducts; or tumor invasion of one secondary bile duct into the contralateral portal vein or hepatic artery. Regional lymph nodes (N); NX: Regional lymph node metastasis cannot be determined; N0: No regional lymph node metastasis; N1: 1 to 3 regional lymph nodes metastases; N2: Metastases in ≥4 regional lymph nodes; Distant metastasis (M); M0: No distant metastasis; M1: Distant metastases (including lymph node metastases beyond the hepatoduodenal ligament and the area behind the pancreatic head and duodenum).

**Table 4:** MSKCC T staging

Staging	Definition
T1	Tumor involving biliary confluence +/- Unilateral extension to the root of the second bile duct.
T2	Tumor involving biliary confluence +/- Unilateral extension to secondary bile duct root and ipsilateral portal vein involvement +/- ipsilateral liver lobe atrophy.
T3	Tumor involving biliary confluence+Bilateral extension to secondary bile duct roots/unilateral extension to secondary bile duct root and contralateral portal vein/unilateral extension to secondary bile duct root with contralateral hepatic lobe atrophy; Main portal vein or bilateral portal vein involvement.

**Table 5:** Gazzaniga Staging

Staging	Definition (Invaded blood vessels)
I	No vascular invasion
II	Unilateral hepatic artery+portal vein
IIIa	Unilateral hepatic artery+portal vein bifurcation
IIIb	Proper hepatic artery+Unilateral portal vein
IV	Proper hepatic artery+portal vein bifurcation

**Table 6:** JSHBPS Staging

Staging	0	I	II	IIIA	IIIB	IVA	IVB
T	TIS	1	2	3	1-3	4	random
N	0	0	0	0	1	random	random
M	0	0	0	0	0	0	1

Note: Tumor size (T); TX: The primary tumor cannot be evaluated; T0: No evidence of primary tumor; Tis: Carcinoma *in situ*; T1a: Tumor confined to mucosa; T1b: Tumor confined to the myofiber layer; T2a: Tumor invades the adipose tissue outside the bile duct wall; T2b: Tumor invades adjacent liver parenchyma; T3: Tumor invades one branch of the portal vein or hepatic artery; T4a: Tumor invades bilateral secondary bile ducts; T4b: The tumor invades the main portal vein or its bilateral branches; or the common hepatic artery, the proper hepatic artery or its bilateral branches; or one secondary bile duct and the contralateral portal vein or hepatic artery. Regional lymph nodes (N); NX: Regional lymph node metastasis cannot be determined; N0: No regional lymph node metastasis; N1: Regional lymph node metastases (group 12, 8, and 13a lymph nodes); Distant metastasis (M); M0: No distant metastasis; M1: Distant metastases (including lymph node metastases beyond the hepatoduodenal ligament and the area behind the pancreatic head and duodenum).

**Table 7:** Mayo staging

Variables	I	II	III	IV
Mass lesion	Unicentric ≤3 cm	Unicentric≤3cm	Unicentric >3cm or multicentric	NA
Metastasis	-	-	+	Peritoneal (or another organ) metastasis
Vascular encasement	-	+	NA	NA
ECOG status	0	1-2	0-2	3-4
CA 19-9 level (U/ml)	<1000	<1000	≥1000	NA
